# Neurocognitive effects of binge drinking on verbal episodic memory. An ERP study in university students

**DOI:** 10.3389/fphar.2023.1034248

**Published:** 2023-02-07

**Authors:** Socorro Rodríguez Holguín, Rocío Folgueira-Ares, Alberto Crego, Eduardo López-Caneda, Montserrat Corral, Fernando Cadaveira, Sonia Doallo

**Affiliations:** ^1^ Department of Clinical Psychology and Psychobiology, Universidade de Santiago de Compostela (USC), Santiago de Compostela, Spain; ^2^ Psychological Neuroscience Laboratory (PNL), Research Center in Psychology (CIPsi), School of Psychology, University of Minho, Campus de Gualtar, Gualtar, Portugal

**Keywords:** alcohol, binge drinking, university students, verbal episodic memory, event-related brain potentials (ERP)

## Abstract

**Background:** Verbal memory may be affected by engagement in alcohol binge drinking during youth, according to the findings of neuropsychological studies. However, little is known about the dynamics of the neural activity underlying this cognitive process in young, heavy drinkers.

**Aims:** To investigate brain event-related potentials associated with cued recall from episodic memory in binge drinkers and controls.

**Methods:** Seventy first-year university students were classified as binge drinkers (32: 17 female) or controls (38: 18 female). The participants completed a verbal paired associates learning task during electroencephalogram (EEG) recording. ERPs elicited by old and new word pairs were extracted from the cued-recall phase of the task by using Principal Component Analysis. Subjects also performed a standardized neuropsychological verbal learning test.

**Results:** Two of the three event-related potentials components indicating old/new memory effects provided evidence for anomalies associated with binge drinking. The old/new effects were absent in the binge drinkers in the two subsequent posterior components, identified with the late parietal component and the late posterior negativity The late frontal component revealed similar old/new effects in both groups. Binge drinkers showed similar behavioural performance to controls in the verbal paired associates task, but performed poorly in the more demanding short-term cued-recall trial of a neuropsychological standardized test.

**Conclusion:** Event-related potentials elicited during a verbal cued-recall task revealed differences in brain functioning between young binge drinkers and controls that may underlie emergent deficits in episodic memory linked to alcohol abuse. The brain activity of binge drinkers suggests alterations in the hippocampal - posterior parietal cortex circuitry subserving recognition and recollection of the cue context and generation of the solution, in relation to verbal information shallowly memorised.

## Introduction

The high prevalence of alcohol binge drinking (BD) among adolescents and young adults is a public health problem worldwide. This pattern of alcohol consumption is particularly prevalent in American, European and Western Pacific countries, where more than 20% of the population between 15 and 24 years of age engage in BD or heavy episodic drinking, according to the World Health Organization (WHO) terminology (Poznyak and Rekve, 2018).

Engaging in BD at young ages is considered one of the leading risk factors for alcohol use disorder (AUD) (Hingson and Zha, 2009; Silins et al., 2018). According to both animal and human studies, it has a significant deleterious effect on neurodevelopment and neurocognitive function (Spear, 2018), particularly related to the intermittent character of BD (i.e., intoxication followed by withdrawal periods) (Crews and Nixon, 2009; Maurage et al., 2020). The prefrontal cortex and the hippocampus are particularly sensitive to the effects of alcohol abuse and dependence, and functions relying on these regions, such as inhibitory control and working memory or declarative/episodic memory, are known to be impaired in people with AUD (Sullivan, 2017).

Medial temporal lobe and prefrontal damage have been robustly associated with deficits in AUD in regard to episodic memory (White et al., 2000; Chanraud et al., 2009). In addition, the late development of these brain regions makes them particularly sensitive to the effects of BD during adolescence and young adulthood (Meda et al., 2018; Pfefferbaum et al., 2018; Walker et al., 2021).

Episodic memory refers to the conscious recollection of events, and it involves encoding, storing, consolidating and subsequently retrieving information. Carbia and others reviewed neuropsychological studies published between 2000 and 2016 and involving episodic memory in adolescent/young adult binge drinkers (BDs) (Carbia et al., 2018). These researchers observed that regarding verbal memory, studies using list learning tasks reported memory difficulties related to executive dysfunctions in BDs and that studies using story learning tasks reported poorer verbal memory (free immediate and delayed recall) in BDs than in controls. Regarding visuospatial memory, the researchers found scant evidence that BDs exhibit difficulties in performing high-demand visuospatial memory tasks. In a meta-analysis of neuropsychological studies, Lees and others (Lees et al., 2019) concluded that there were no significant effects regarding deficits in episodic memory (immediate, recent, long-term) related to BD, although they also highlighted a longitudinal study (Carbia et al., 2017) that linked BD with deficits in immediate and delayed recall that remained over a 6-year follow-up period in stable BDs. A more recent study on BDs reported poorer delayed recall of verbal and visual memory in standardized neuropsychological tests (Kang and Kim, 2022).

Measures of brain activity may be sensitive to slight neurocognitive dysfunction in non-pathological populations. The small number of studies that have used electroencephalography (EEG)/event-related potentials (ERPs) or functional magnetic resonance imaging (fMRI) to assess declarative memory in adolescent/young adult BDs is surprising, as such studies can be used in a complementary approach to neuropsychological testing and provide some insight into underlying neural activity during memory processing.

Both fMRI and ERP recording are useful techniques for studying declarative memory, although they are not without limitations. fMRI studies provide information about the neural regions involved in different memory types, systems or processes (implicit or explicit; semantic and episodic; recollection or recognition/familiarity; encoding, consolidation and retrieval) (Ranganath and Ritchey, 2012; Squire et al., 2015; Kim, 2019; Palacio and Cardenas, 2019). EEG, particularly ERPs, which have excellent temporal resolution, provide information about the dynamics of memory processing (Staresina and Wimber, 2019).

Brain activity associated with retrieval from declarative episodic memory is usually studied using tasks in which subjects are asked to recognize or recall previously studied information. Brain activity is compared between old items (previously memorised) and new items (not previously studied). Old and new items elicit differential brain activity as recorded by ERPs, referred to as old/new effects.

Regarding recognition tasks, most studies have described the FN400 effect (or midfrontal old/new effect, peaking at around 400 ms after the retrieval cue, exhibiting larger negativity for new items), identified as an index of familiarity in recognition task, and the late parietal component (LPC), or parietal old/new effect (with an onset around 400–500 ms after the cue: larger for old items and when retrieval of contextual information is required), which is considered an index of recollection (Friedman and Johnson, 2000; Rugg and Curran, 2007; Rivas-Fernández et al., 2020). Other components that appear even later, such as the right frontal old/new effect (or right frontal episodic memory effect, extending from 500 to 2000 ms) and the late posterior negativity (LPN) (which is larger for old than new items and peaks after the subject’s response), have been related to action monitoring and post-retrieval processing during reconstruction of the study episode (Friedman and Johnson, 2000; Cruse and Wilding, 2009; Mecklinger et al., 2016; Rivas-Fernández et al., 2020).

In cued-recall tasks, in which subjects are asked not only to recognise the item, but also to generate the solution, the cue elicits a broad wave extending from 300–500 to several hundred milliseconds over frontal, central and parietal areas. This wave is more positive for deeply than shallowly encoded items and when contextual information is also recalled. It is composed of several overlapping subcomponents, some of which are common to recognition tasks (LPC and right frontal episodic memory effects, LPN) and others specific to cued-recall (left inferior prefrontal activity, presumably related to the need to generate a solution) (Allan and Rugg, 1998; Allan et al., 2000; Friedman and Johnson, 2000; Mecklinger et al., 2016).

Study of the brain activity associated with retrieval of episodic memory in binge drinkers is scarce, although it is considered useful both for characterising the neural activity associated with memory processes (Wilding and Ranganath, 2012) and for detecting abnormalities related to alcohol consumption (Almeida-Antunes et al., 2021). As far as we know, only one study, by Smith and cols (Smith et al., 2017) addressed this issue. These researchers recorded ERPs during retrieval in a delayed recognition trial of an adaptation of the Rey Auditory Verbal Learning Test (RAVLT), identifying two ERP components showing old/new effects: a frontal negative component (N340) and a parietal positive component (P540). Both of these showed a similar old/new effect in controls and BDs, but overall P540 was larger in BDs than in controls.

In the present study, we aimed to extend the research on possible anomalies in brain activity during memory retrieval associated with early alcohol consumption by examining the ERPs elicited during cued-recall episodic memory retrieval in a sample of young BDs and controls. We adapted the verbal paired associates task used by Schweinsburg and others (Schweinsburg et al., 2010, 2011) for ERP recording, to measure electrical brain activity elicited by cued-recalled old (previously memorised) and new (seen only once) word pairs. This assessment was supplemented by measuring immediate cued-recall in a standardized neuropsychological test of verbal learning. Subjects were evaluated during their first year at university. A small subsample of the subjects were followed up 2 years later, and the data obtained, which were analysed for exploratory purposes, are reported in Supplementary Material S1.

We hypothesized that the demonstrated effect of alcohol binge drinking on prefrontal and medial temporal brain regions in young adults should be reflected in anomalous brain activity during retrieval of episodic memory, even in the absence of behavioural performance impairment. This hypothesis is based on previous studies showing abnormalities in brain activity during cognitive function despite normal behavioural performance in young, non-AUD binge drinkers; these results have been interpreted as a sign of a subtle deleterious effect of binge drinking on brain functioning, which may be compensated by increased brain recruitment to achieve normal performance in the early stages of abuse, and which would progress, if persistent, to impaired behavioural performance characteristic of the AUD (see, for example, Lannoy et al., 2019).

These anomalies are expected to be observed particularly in ERP components related to memory subprocesses that are most dependent on executive functions, such as recollection, action monitoring and post-retrieval processes, as executive functions have been indicated to be the cognitive functions most severely affected by early alcohol consumption (Carbia et al., 2018; Lannoy et al., 2019). We did not formulate *apriori* hypotheses about the influence of sex on the relationship between BD and brain activity during memory retrieval, because previous research does not indicate a clear role of this variable (Carbia et al., 2018; Almeida-Antunes et al., 2021). Thus, this factor will be considered in accordance with the recommendations by Joel and Fausto-Sterling, 2016.

## Materials and methods

### Participants

A sample of 70 first-year students (18–19 years old) from the University of Santiago de Compostela (Spain) participated in this study, which forms part of a broader research project on binge drinking among university students and has been approved by the Bioethics Committee of the University. Initially, 1,328 volunteers participating in the epidemiological phase of the research completed a classroom-administered questionnaire composed of the Alcohol Use Disorders Identification Test (AUDIT) (Saunders et al., 1993), with additional questions regarding consumption of alcohol and other substances (illegal drugs and medications) and also sociodemographic information for epidemiological purposes (such as type of household or parents’ level of education).

The students were screened on the basis of their responses to the questionnaire, and 200 of the respondents, who fulfilled the initial selection criteria, and who also provided contact information, were interviewed. These students signed informed consent and received compensation (10 euros) for their participation. The semi-structured interview included the Mini International Neuropsychiatric Interview, Spanish version 5.0.0 (Ferrando et al., 2000), and the Symptom Checklist-90-R (SCL-90-R) (Derogatis, 1983) for assessing history or current psychopathological symptomatology. It also included a detailed questionnaire about substances use, based on the Cannabis Abuse Screening Test (CAST) (Cuenca-Royo et al., 2012), the Nicotine Dependence Syndrome Scale, short version (NDSS-S) (Shiffman et al., 2004) and a diary of alcohol consumption based on the revised version of the Alcohol Use Questionnaire proposed by Townshed and Ducka (Townshend and Duka, 2002).

Selection for neurocognitive assessment was based primarily on the drinking pattern. Subjects with an alcohol consumption of six or more drinks per episode at least once a month (AUDIT item 3) and a drinking rate of at least three drinks per hour in these episodes were selected as binge drinkers; this criterion was defined to approximate the level of consumption to the NIAAA definition of binge drinking (National Institute on Alcohol Abuse and Alcoholism, 2016). Subjects were included in the control group if they never partook in 6-drink episodes and never drank more than two drinks per hour. Inclusion and exclusion criteria, applied on the basis of information obtained in the interview, are summarised in Table 1.

**TABLE 1:** Inclusion and exclusion criteria for sample selection, based on interview.

Inclusion criteria	Exclusion criteria
• First-year university students (18–19 years)	• Personal or family (first-degree) history of major psychopathological disorders (DSM-IV), including alcohol or substance abuse
• **Binge drinkers:** ≥ 6 alcoholic drinks/occasion at least once a month and speed of consumption ≥3 alcoholic drinks/hour	• History of traumatic brain injury or neurological disorder
• **Control group:** < 6 alcoholic drinks/occasion and speed of consumption ≤2 drinks/hour	• Any episode of loss of consciousness >20 min
	• Use of illegal drugs (except infrequent cannabis^a^)
	• Use of psychoactive medication
	• Uncorrected sensory deficits
	• AUDIT score ≥20

^a^ Less than once per week.

Seventy-four subjects (100% Caucasian) who fulfilled these criteria consented to take part in the neurocognitive assessment. These participants received 20 euros for their collaboration. Four of the participants were later excluded because of the low quality of the EEG recording. Of the final 70 subjects, 32 (17 females) were included in the binge-drinking (BD) group and 38 (18 females) in the control (CN) group. Demographics and substance use characteristics are summarised in Table 2.

**TABLE 2:** Demographic and substance use characteristics of the sample (mean ± standard deviation).

	Controls	Binge drinkers
N (females)	38 (18)	32 (17)
Age [range]	18.5 ± 0.3 [18-19]	18.4 ± 0.3 [18-19]
Age of drinking onset	16.83 ± 1.46	15.21 ± 1.21
Total grams of alcohol in a standard drinking episode^a^	10.59 ± 13.44	109.45 ± 30.14
Speed of consumption: drinks/hour^a^	0.25 ± 0.34	3.38 ± 0.75
Estimated BAC in a standard drink episode^b^ ^,^ ^a^	0.006 ± 0.01	0.23 ± 0.08
Percentage of times became drunk when drinking^a^	0.27 ± 1.64	39.69 ± 25.14
Total AUDIT score^a^ [range]	0.95 ± 1.48 [0-6]	9.22 ± 2.51 [4-14]
N regular tobacco smokers^c^	0	4
SCL-90-R - GSI (percentile scores)	22.36 ± 21.76	34.19 ± 29.98

^a^ Student t-test *p* < 0.05.

^b^ Gr/dL (calculated using the classic Widmark formula (Widmark, 1932; see in Kelly and Mozayani, 2012).

^c^ Daily (max. Five cigarettes/day); SCL-90-R - GSI: Symptom checklist-90-revised - global severity index; AUDIT: alcohol use disorders identification test.

### Procedure

Participants were asked to abstain from consuming alcohol and other drugs (including medical prescriptions) for at least 24 h before the electrophysiological assessment, and to sleep for at least 7 hours the night before testing. An alcohol breath level of 0.00% was verified by breathalysing the participants on arrival at the laboratory.

The electrophysiological assessment began with the verbal paired associates task, which was an adaptation to ERP recording of the task used in fMRI studies by Schweinsburg and others (Schweinsburg et al., 2010, 2011).

During the EEG recording, subjects were seated on an armchair inside an electrically shielded, dimly lit, sound-attenuated room. The subjects were instructed not to move during EEG recording, to use the pauses programmed in the task to adjust their position, and to fix their gaze on a small cross in the centre of the screen (a 20″CRT monitor, 1,152 × 864 pixels, refresh rate 85 Hz), located 100 cm in front of their eyes.

The subjects were asked to learn and recall a series of phonetically related pairs of two-syllable nouns, each displayed for 2 s in the centre of the screen. A total of 192 words were selected from the *BuscaPalabras* (B-Pal) base vocabulary, which includes 31,491 Spanish words (Davis and Perea, 2005).

The task involved first memorizing a list of 16 word pairs, which were presented sequentially in the centre of the screen (learning block); immediately after this series, recall was tested by presenting the first noun of each pair and asking subject to verbalize the second one (or to say ‘I don’t remember’) when a question mark appeared on the screen (recall block). The order of presentation of the words varied between the learning and the recall blocks. The series (learning and recall blocks) were presented twice, ensuring subjects correctly recalled at least 10 of the 16 pairs (none of the subjects required presentation of a third block to reach this threshold).

Participants subsequently performed a new series (old/new learning and recall blocks), composed of 32 word pairs: the 16 previously memorised pairs (old pairs, OP) intermixed with 16 novel pairs (new pairs, NP). EEGs were recorded during both the learning and the recall blocks.

**FIGURE 1:**
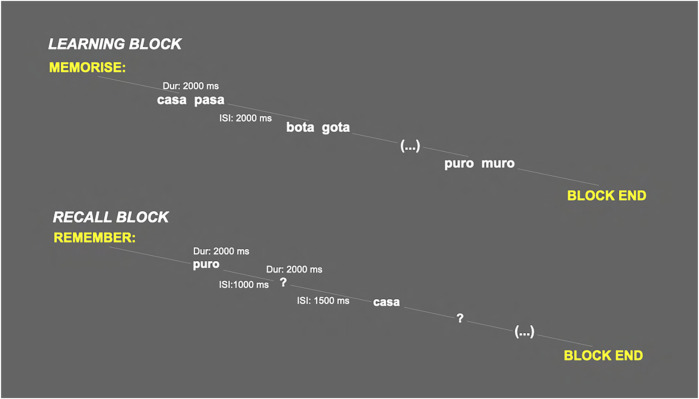
Schematic representation of the learning and recall blocks of the verbal paired associates task (Dur: stimulus duration; ISI: interstimulus interval).

This task was repeated three times, with three different lists of words, yielding a total of 48 OP and 48 NP. The complete task lasted approximately 50 min, including pauses between blocks autoregulated by the participant. The parameters of the task (stimuli duration and interstimulus intervals) are summarised in Figure 1.

In a separate session, these participants also underwent a neuropsychological assessment including the TAVEC (*Test de aprendizaje verbal España-Complutense*) (Benedet and Alejandre, 2014), a verbal episodic memory test based on the California Verbal Learning Test (CVLT) (Delis et al., 1987).

This test began with the examiner reading the learning list (List A, 16 items) and asking the participant, immediately after the reading, to remember as many words as possible. This procedure was repeated 5 times (learning trials 1–5). The examiner then read the interference list (List B, 16 items) and asked the subjects again to freely recall as many of the words on this list as possible. Free recall of List A was then requested (short-term free recall trial), followed by a cued recall test, with the semantic category of the words (tools, fruits, clothes and spices) as cues (short-term cued recall trial). Twenty minutes later, during which subject were carrying out other neuropsychological tests (none of them on declarative memory or vocabulary), the test was resumed: the participants were asked to freely recall items from List A (long-term free recall trial) and then to perform a cued-recall test of the same list (long-term cued recall trial). Finally, a recognition test was applied: a list of 44 items (16 from the List A, eight from the List B and 20 phonologically or semantically similar new words) was read to the participant, who had to say, after each word, whether it belonged to List A or not (recognition trial).

### EEG data acquisition and processing

The EEG was recorded during the old/new learning and recall blocks of the verbal paired associates memory task. Electrodes were located using a BrainCap with 32 positions according to the extended 10-20 International System (Nuwer et al., 1998). Active scalp electrodes were referred to the nose tip, and ground electrode was placed at Fpz. Electrooculogram was recorded from vertical and horizontal bipolar channels to control for eye movements and blinks. Electrode impedances were kept below 20 kΩ. EEG signals were continuously amplified and digitized, at a sampling rate of 500 points/s, and filtered on-line with a 0.1–100 Hz band pass filter and a 50 Hz notch filter.

EEG data were off-line processed with BrainVision Analyzer software (Version 2.0) to obtain the event-related potentials (ERP) elicited by the word pairs in the old/new recall blocks. Ocular artefacts were corrected by the procedure developed by Gratton and others (Gratton et al., 1983). The EEGs recorded during the old/new recall blocks were digitally filtered offline with a 0.1–12 Hz and segmented into epochs of 2,200 ms, from 200 ms pre-stimulus to 2000 m post-stimulus. This temporal interval was chosen for analysing effects related to memory recall reported in the literature, with latencies of 1,400 ms sometimes exceeded (Friedman and Johnson, 2000). After adjusting the pre-stimulus baseline period to 0 μV, epochs exceeding ± 80 μV were rejected.

EEG epochs were then averaged separately for old pairs (OP) and new pairs (NP). The resulting averaged ERPs comprised at least 17 epochs for each subject and type of stimulus (mean ± SD: 34.6 ± 6.8 for old pairs, 27.0 ± 6.6 for new pairs, without differences between groups); as indicated in the participants subsection, four subjects were excluded for this reason.

ERPs for each subject and type of stimulus (OP, NP) were then processed with Dien’s *ERP PCA toolkit (v.2.96)* (Dien, 2010b) to isolate overlapping components of the ERPs related to memory processing. The decomposition was conducted in two-step sequential temporospatial Principal Component Analysis (PCA) (Dien, 2010a).

After reducing the sampling rate to 250 points/s, the data were subjected to a temporal Promax rotation using the voltage EEG values (550 samples) as variables, and electrode sites (32), types of stimulus (2) and subjects (70) as observations. Thirteen temporal factors were extracted using the parallel test (Horn, 1965) of the scree plot (Cattell, 1966). In a second step, these factors were then subjected to a spatial Infomax (ICA) rotation with the 32 scalp recording sites as variables and the temporal factor scores, types of stimulus and subjects as observations. Two spatial factors were extracted from each temporal factor, resulting in 26 temporospatial factors explaining 90% of the variance.

These factors were then rescaled to microvolts to facilitate interpretation, and values of amplitude at the peak channel and the peak time point were automatically extracted for each of the 16 factors accounting for a minimum of 0.5% of the variance. The characteristics of these factors (ERP components) are summarised in Table 3.

**TABLE 3:** Temporospatial factors (ERP components), isolated *via* two-steps temporospatial PCA, explaining at least 0.005 of the variance.

Factor	Peak latency (ms)	Peak channel (polarity)	Explained variance
TF01SF1	1,656	Oz (-)	0.392
TF01SF2	1,656	AF3 (+)	0.012
TF02SF1	812	POz (-)	0.170
TF02SF2	812	O1 (+)	0.015
TF03SF1	408	Pz (+)	0.100
TF03SF2	408	Fz (-)	0.025
TF04SF1	1,260	POz (-)	0.055
TF05SF1	624	POz (+)	0.028
TF06SF1	200	PO7 (-)	0.015
TF07SF1	280	POz (+)	0.010
TF08SF1	1976	Pz (+)	0.013
TF09SF1	1,048	PO3 (-)	0.010
TF10SF1	340	Pz (-)	0.009
TF11SF1	152	PO7 (+)	0.008
TF12SF1	96	O2 (+)	0.009
TF13SF1	1,440	PO4 (-)	0.009

### Statistical analysis

Statistical analysis of the ERP components was conducted using robust analysis of variance (ANOVA) implemented in the ERP PCA Toolkit (Keselman et al., 2003; Dien, 2010b), with Welch-James approximate degrees of freedom based on 499,999 bootstrap samples and a familywise corrected alpha of 0.05 (TWJt/c). The Dunn-Šidák correction for multiple comparisons was applied to post-hoc pairwise contrasts.

The ERP components were subjected to preliminary analysis in order to determine which would be selected as indicators of old/new effects (a difference in amplitude between the previously memorised and the new word-pairs). For this purpose, the two types of stimulus (OP vs. NP) were compared in the 16 ERP components selected above.

Another preliminary analysis was carried out to decide whether to include Sex as a main factor (independent variable), following the recommendation that, in absence of *a priori* hypothesis, it must be included only when interacts with the main variables of the research (Joel and Fausto-Sterling, 2016). With this aim, a Group x Sex x Type of stimulus analysis was performed on each of the ERP components that showed old/new effects.

Given the exploratory nature of these preliminary analyses, familywise correction was not applied, and factors were considered in the main design even though the alpha level was at the borderline of the significance threshold.

The main analyses were then conducted to address the primary focus of this research (memory retrieval), taking the results of these exploratory analyses into account. The ERP components indicating a memory old/new effect were examined considering the within-subjects factor Type of stimulus (OP vs. NP) and the between-subjects factor Group (CN vs. BD), and where relevant, Sex (male vs. female). The dependent variables were the voltage of the ERP components isolated by PCA (at the peak channel and time point).

In addition, related behavioural variables were analysed using IBM-SPSS (v.25): Percentage of hits to OP and NP in the old/new recall blocks of the task were analysed using a conventional mixed-model 2 × 2 ANOVA (Group x Type of stimulus), with an alpha threshold of 0.05, and post-hoc pairwise comparisons with Bonferroni correction. The TAVEC, short-term cued-recall scores (range 0–16) from the two groups wer compared by a Student t-test; this variable was selected because it was the TAVEC measure closest to the task used with ERPs.

## Results

### Preliminary analysis

**FIGURE 2:**
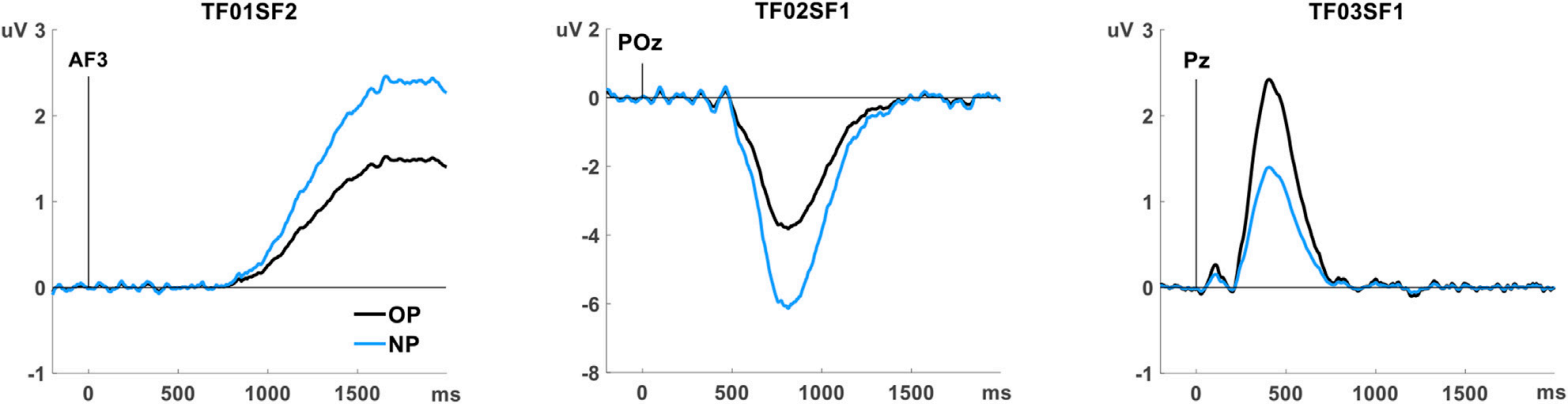
Temporospatial factors (ERP components) showing old/new memory effects, rescaled to µV at the channel with the peak amplitude. Grand average for the old word pairs (OP, black line) and the new word pairs (NP, blue line).

Preliminary analysis conducted to identify the ERP components associated with memory revealed three ERP components with significant old/new effects. One of them (TF03SF1) did not withstand the correction for multiple comparison recommended when there are *a priori* no criteria for selecting the factors. Non-etheless, it was selected for subsequent analysis because its latency and topography were consistent with previously described ERP memory components. These components are summarised in Table 4 and illustrated in Figure 2.

**TABLE 4:** Temporospatial factors (ERP components) with old/new effect selected for main analysis.

Factor	Peak latency (ms)	Peak channel (polarity)	Old/new effect		Trimmed means (SE) [µV]	
			t	*p*	OP	NP
TF01SF2	1,656	AF3 (+)	16.16	0.00057	1.46 (0.03)	2.42 (0.03)
TF02SF1	812	POz (-)	15.71	0.00015	-3.53 (0.10)	-6.08 (0.10)
TF03SF1	408	Pz (+)	7.07	0.00963^a^	2.35 (0.09)	1.18 (0.09)

SE: standard error; OP: old word pair; NP: new word pair.

^a^ Does not fulfil familywise corrected alpha criteria (Dunn-Šidák correction): .0032007.

The earlier component (TF03SF1), peaking at 408 ms with positive polarity at a posterior location (Pz) (P408p), resembles the episodic memory effect linked to recollection (LPC or parietal old/new effect), although with earlier latency than expected. The later components, TF02SF1, with negative polarity at posterior POz (N812p), and TF01SF2, positive at the anterior AF3 (P1656a), are related to the post-retrieval processing on the cue and the generation of the completion (the second word of the pair).

Analysis of these three ERP components by considering the Sex factor revealed an influence of this variable on TF03SF1 (P408p), which showed Sex x Type of stimulus interaction (T_WJt_/c (1.0.62.3) = 4.45, *p* = 0.038, mean-squared error (MSe) = 6.01) and an almost significant Sex × Group interaction (T_WJt_/c (1.0.58.4) = 3.80, *p* = 0.0568, MSe = 65.90). No other ERP components showed interactions including Sex; therefore, this factor was only included in the core analysis of TF03SF1 (P408p); Sex-disaggregated data for the dependent variables are included in the Supplementary Material S1.

#### ERP components

**FIGURE 3:**
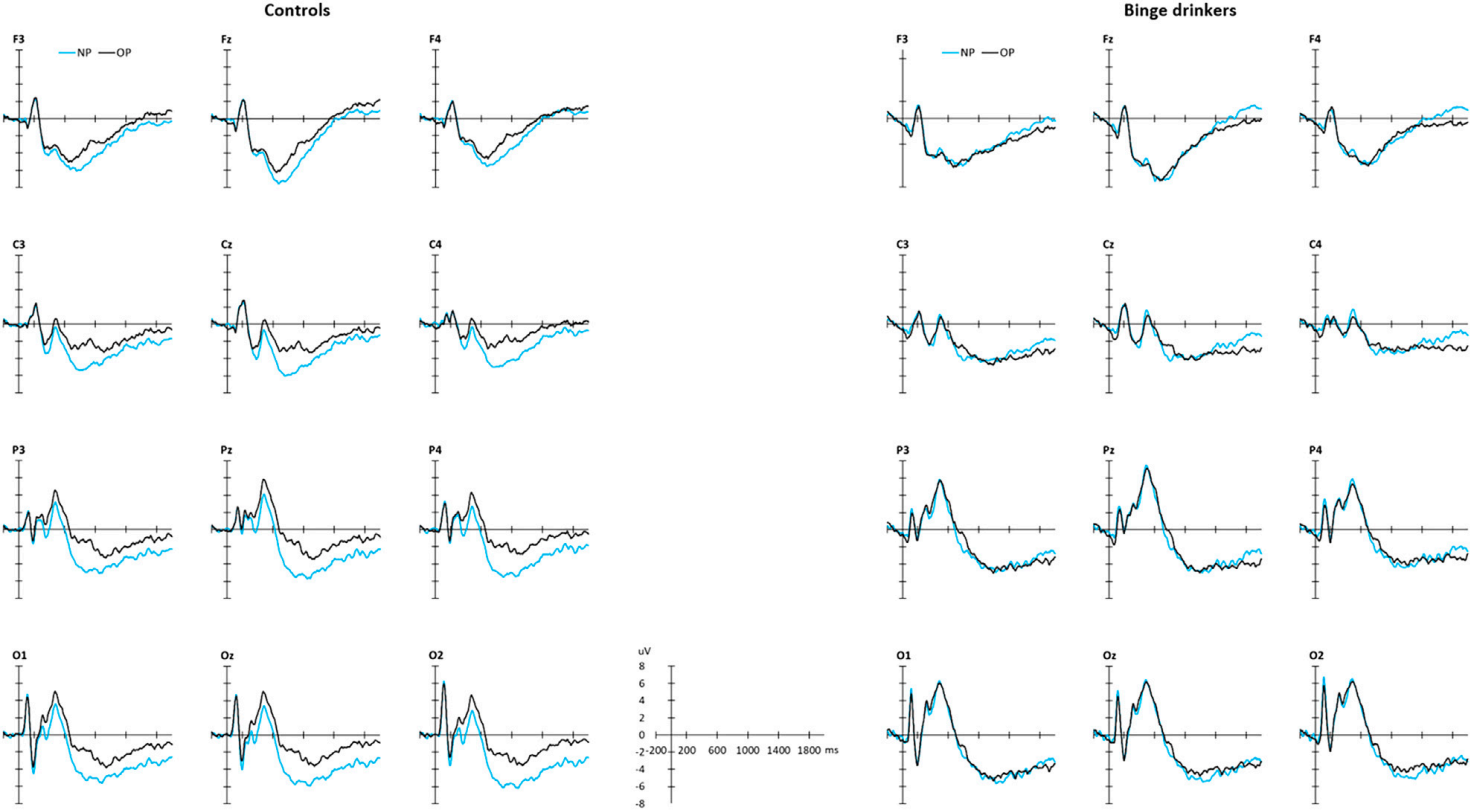
Grand average ERPs elicited by old word pairs (OP, black line) and new word pairs (NP, blue line), in a set of the recorded sites, in the control (left) and binge drinking (right) groups.

The grand averaged ERP recordings are shown in Figure 3, and the descriptive statistics (mean amplitude and standard deviation) of the selected ERP components (factors rescaled to microvolts at the peak latency and electrode site), for each group (binge drinkers and controls) and type of stimulus (old and new word-pairs) are summarised in Table 5.

**TABLE 5:** Mean (standard deviation) amplitude of the selected ERP components (factors rescaled to µV at the peak latency and electrode site) for each group (binge drinkers and controls) and type of stimulus (old and new word pairs, OP/NP) (ordered by latency).

Factor/ERP component	Controls		Binge drinkers	
	OP	NP	OP	NP
TF03SF1/P408p	1.78 (5.97)	0.07 (5.51)	3.08 (6.29)	2.94 (6.20)
TF02SF1/N812p	-3.54 (7.12)	-7.04 (7.25)	-4.05 (7.10)	-4.91 (6.31)
TF01SF2/P1656a	1.38 (2.02)	2.31 (2.00)	1.60 (2.34)	2.57 (2.14)

P408p was analysed by including the variable Sex. This analysis revealed a Type of stimulus effect (T_WJt_/c (1.0.62.3) = 5.14, *p* = 0.0268, MSe = 6.01), with larger amplitudes for OP (averaged trimmed mean = 2.37 µV) than NP (1.41 µV); and a Type of stimulus x Group effect (T_WJt_/c (1.0.62.3) = 4.30, *p* = 0.0418, MSe = 6.01), with the old/new effect present in controls (T_WJt_/c (1.0.34.0) = 9.66, *p* = 0.00392, MSe = 6.24), but absent in BDs, and with larger amplitude (T_WJt_/c (1.0.57.8) = 4.29, *p* = 0.043, MSe = 34.19) for NP in BD (2.89 µV) than CN (-0.06 µV). The Type of stimulus × Sex interaction (T_WJt_/c (1.0.62.3) = 4.45, *p* = 0.039, MSe = 6.01) was also significant, with the old/new effect only emerging in females (T_WJt_/c (1.0.32.0) = 8.56, *p* = 0.0068, MSe = 6.95).

N812p also presented a Type of stimulus effect (T_WJt_/c (1.0.62.0) = 14.89, *p* = 0.00028, MSe = 12.46) with larger negative amplitude for NP (-5.97 µV) than OP (-3.59 µV), and a Type of stimulus × Group interaction (T_WJt_/c (1.0.62.0) = 4.11, *p* = 0.0457, MSe = 12.46), showing that the old/new effect was present in controls (T_WJt_/c (1.0.35.0) = 18.89, *p* = 0.000048, MSe = 12.57), but absent in BDs.

Group by Type of stimulus statistical analysis of the P1656a amplitude revealed a significant main effect of Type of stimulus (T_WJt_/c (1.0.59.3) = 15.99, *p* = 0.00086, MSe = 1.92), with larger amplitude in response to NP (2.44 µV) than to OP (1.47 µV). There were no Group or Type of stimulus x Group effects.

#### Behavioural performance

Descriptive estatistics of the behavioural measures are summarized in Table 6. The percentage of hits to old and new word pairs showed, as expected, a Type of stimulus effect (F (1,68) = 265.947, *p* < 0.0001, ƞ^2^_p_ = 0.796), which was more accurate for OP (91.90%) than NP (67.14%), but there were no differences between groups or Type of stimulus × Group interaction.

**TABLE 6:** Descriptive statistics on the behavioural measures. Percentage of hits in the verbal paired associates task (old and new word pairs, OP/NP) and scores in the Short-term cued-recall measure of the TAVEC. Mean (standard deviation) for each group.

	Controls		Binge drinkers	
	OP	NP	OP	NP
Verbal memory task % hits	91.67 (8.36)	66.72 (16.80)	92.19 (6.39)	67.64 (15.55)
TAVEC	13.92 (1.78) [11-16]		12.91 (1.84) [9-16]	
Short-term cued-recall score [range 0–16]				

Regarding the TAVEC measure (short-term cued-recall score), a Group effect was observed (t (68) = 2.344, *p* = 0.022), with poorer performance in the BD (12.91) than in the CN group (13.92). Finally, we should point out that, although the scores for free recall or long-term recall were not considered here, because they were not a direct counterpart of the verbal paired associates task, we analysed them for informative purposes, and did not find any other effects associated with alcohol consumption.

## Discussion

ERPs elicited by old and new word-pairs during immediate cued-recall in young binge drinkers and controls were identified in this research by temporospatial Principal Component Analysis (Dien, 2010a; 2010b), bearing in mind the variability of the ERP components and subcomponents associated with memory retrieval across task paradigms, and the overlap between these (see Friedman and Johnson, 2000). With this approach, we found three ERP components that indicated old/new effects and resembled, in terms of latency, polarity and topography, the components already described in the relevant literature. The results showed that the old/new effects observed in the control group were absent in the binge drinkers in the two subsequent posterior components, identified as the late parietal component (LPC) and the late posterior negativity (LPN), whereas the late frontal component revealed similar old/new effects in both groups.

The literature on declarative episodic memory describes frontal (FN400) and parietal (LPC) ERP components, at latencies of between 400 and 800 m (variable across tasks), with old/new effects, which have been associated with familiarity (or priming) and recollection, respectively, in recognition tasks. Although more controversially, the LPC has also been described in cued-recall tasks (Friedman and Johnson, 2000; Fay et al., 2005). In the present study, a large parietal positive waveform resembling this LPC was observed, peaking at around 500 ms (see Figure 2). The PCA analysis allowed us to isolate the ERP component named P408p, peaking at Pz, that showed the expected old/new effect (larger positive amplitude for old than for new word pairs).

We identified two other components, of longer latency, showing old/new effects: N812p (with negative polarity, peaking at 812 ms at POz), which was less negative for old than for new word pairs, and P1654a (with positive polarity, peaking at 1,656 ms at AF3), which had more positive amplitude for new than for old word pairs.

The N812p is consistent with the late posterior negativity (LPN) described in ERP, memory-related studies. Initially reported in recognition tasks, it has also been observed in cue-recall tasks involving both episodic memory (Bai et al., 2015) and semantic memory (Hellerstedt and Johansson, 2016), with a broad latency range, from 700-800 to 1,000–1,200 m, and a possible origin in the medial posterior parietal cortex. Although described as more negative for old than for new items in recognition tasks (Mecklinger et al., 2016), some studies using cued-recall paradigms have reported less negative LPN for old items than for new ones, as well as for deeply processed than for shallowly processed items, and for more accurately than for less accurately remembered items (Fay et al., 2005; Bai et al., 2015; Hellerstedt and Johansson, 2016), as in the case of the N812p in this study.

The P1656a resembled the long-lasting frontal old/new effects in ERPs associated with postretrieval processes, such as the (right) frontal old/new effect described in recognition tasks (Wilding and Rugg, 1996) and the left inferior prefrontal activity elicited in cued-recall tasks (Johnson et al., 1998). These components vary according to the task features and the material that has to be retrieved, but share a frontal distribution, according to the role of the prefrontal cortex in postretrieval control operations, and a late latency, usually beginning after the LPC and lasting several hundreds of milliseconds, until the end of the epoch (Wilding and Ranganath, 2012).

The LPN and the late frontal old/new effects are considered indexes of postretrieval processes, both mnemonic (to generate a solution and form an integrated representation of a prior episode), and non-mnemonic (control and monitoring operations to assess the retrieved information in the context of goal-directed behaviour) (Wilding and Ranganath, 2012; Mecklinger et al., 2016).

Comparison of these ERP components in relation to alcohol consumption revealed anomalies in BDs. First, the LPC-like component (P408p) showed group-related differences, as the old/new parietal effect appeared to be influenced by alcohol consumption. The effect was present in controls but absent in BDs, in whom new word pairs elicited an equally high voltage as elicited by old word pairs. Moreover, this effect was qualified by the Sex x Type of stimulus interaction: the old/new effect only reached statistical significance in female controls.

As explained above, the LPC, or Parietal old/new effect, has been considered a correlate of recollection and described as more prominent when correctly retrieving contextual (episodic) information or when deeper processing is required on encoding (Friedman and Johnson, 2000; Rugg and Curran, 2007). The results obtained with BD subjects in this study indicated an abnormally large amplitude of the LPC for new pairs (relative to controls), which would obscure the old/new memory effect. This pattern of greater brain activity (usually referred to as hyperactivation) in BDs than controls during cognitive processing is frequently detected with both ERP and fMRI techniques (Lannoy et al., 2019; Almeida-Antunes et al., 2021), usually in the absence of behavioural impairment, and it has been interpreted as reflecting a compensatory mechanism whereby increased recruitment of neural resources is required to achieve a similar level of performance. In this study, the hyperactivation phenomenon appeared when new (seen once) word pairs are retrieved.

The study reported by Smith et al. (2017) is the most similar for comparative purposes. These researchers identified an LPC-like component that emerged during free-recall of a verbal learning task and that was referred to as P540. Overall the amplitude of this component was larger in BDs, but the old/new effect remained. In our study, although the overall amplitude was also apparently larger in BDs (2.93 ± 0.20 µV) than in controls (0.85 ± 0.17 µV), the difference was not statistically significant; however, we only observed the expected old/new effect in controls (particularly in women) and observed that the amplitude of the LPC-like component was larger for new word-pairs in BDs. Thus, some neural hyperactivation at the time of the brain index of memory recollection was observed in both studies, although our findings seem to be specific to new (once seen) word pairs.

Moreover, we can also compare the present findings with those of our previous study of memory encoding with another sample of BDs and controls, although with a task of a different nature (Folgueira-Ares et al., 2017). In the earlier study we also observed, at similar latencies (350–650 m) but during encoding, that ERPs in BDs did not indicate the memory effect (Dm effect) observed in controls at posterior electrode sites. Considering that episodic memory theories and neuroimaging studies have proposed that there is an overlap between brain activation patterns of encoding and retrieval (Persson and Nyberg, 2000), these findings call for future ERP studies of the two processes in the same samples and tasks, to assess whether these two anomalies observed in BDs can be replicated and related.

As discussed above, the two other ERP components indicating old/new effects in this study were associated with those described in literature as correlates of postretrieval processing. The N812p also indicated an absence of this old/new effect in BDs. Unlike in the case of the P408p/LPC described above, the absence of the memory effect in the N812p did not seem to indicate a hyperactivation phenomenon, as it was due to a lower (although not significant) voltage elicited by new word pairs in BDs (-4.93 ± 0.14 µV) than in controls (-7.00 ± 0.13 µV). The later, left frontal component, P1656a, did not show any differences associated with alcohol consumption. Smith et al. (2017) analysed a shorter ERP epoch than we did in the present study (ending 900 m after the stimulus onset), and therefore these late components did not appear, precluding any comparison.

Our results indicate anomalous brain activity (relative to controls) in posterior regions during the retrieval of new (shallowly memorised) word pairs. The anomalies would consist of increased neural activation during cue recognition, indicated by P408p/LPC, and decreased activity during solution generation, indicated by N812p/LPN. Based on a recent comprehensive review on the neural basis of memory recall (Staresina and Wimber, 2019), these anomalies will depend on medial temporal lobe (MTL) and posterior parietal cortex (PPC) nodes in the episodic memory circuitry. These authors propose that episodic memory would involve a transient signal from the MTL (hippocampus), around 500 ms after the cue, at the time of the old/new parietal effect (LPC), which would initiate cortical reactivation of the memory engram. After 600 ms, the PPC would then respond to this bottom-up hippocampal signal with sustained activity during the maintenance interval (resembling the LPN), to allocate working memory and attentional resources to the retrieval of task-relevant mnemonic features. Although our ERP data do not enable us to gain insight into neural generators, they are consistent with the model derived from different sources of data (EEG, MEG, fMRI, intracranial recordings) and may indicate abnormal activity associated with BD in hippocampus/LTM and in association regions of the PPC. Whether this electrophysiological abnormality is a result of consumption or is an endophenotype that precedes it, is not discernible in the absence of data prior to the onset of BD.

Notably, no anomalies were observed in the frontal P1656a. As stated above, this component would be related to postretrieval processes associated with monitoring of the solution generated. BD has been associated with anomalous brain activity in young adults during executive functions, such as performance monitoring (Smith et al., 2016; Almeida-Antunes et al., 2021), and with impairments in executive functions involved in neuropsychological tests of verbal memory (Carbia et al., 2018). Some effects associated with alcohol consumption on this component were therefore expected.

Finally, complementing these results, the behavioural performance in the verbal paired associates task did not indicate any differences between BDs and controls; this was expected, as most ERP studies reporting anomalies in brain function in BDs have not found impairments in behavioural performance. This has usually been interpreted as a sign that ERPs are sensitive to a slightly deleterious effects of alcohol that can be compensated for (e.g., by recruiting more neural resources) to produce normal performance. Of course, replication studies are needed to confirm these results, as well as follow-up studies to assess the evolution of electrophysiological and behavioural measures with the onset and persistence of BD.

Nonetheless, the neuropyschological test results showed evidence consistent with memory impairment in BDs: In the short-term cued-recall trial of the TAVEC, in which subjects had to recall previously studied words cued by the semantic category, the BD group obtained lower scores. Previous studies with standardised neuropsychological tests consisting of word lists have reported mixed results. Consistent with our results, a study with French university students (Gierski et al., 2020), using a modified version of the Free and Cued Selective Reminding Test (FCSRT), reported that BDs showed marginally lower scores in the immediate recall cued score, which is very similar to our short-term cued-recall score. In a systematic review, Carbia et al. (2018) observed that verbal memory impairments associated with BD mainly appeared in tasks using lists of related words (such as the TAVEC), unlike in tasks using lists of unrelated words (such as the Rey Auditory Verbal Learning Test). These researchers concluded that the impairments would be associated with executive dysfunctions, such as poor semantic clustering, which is more involved in the recall of related than unrelated words. The present findings, with poor performance in the TAVEC and no differences from controls in the verbal paired associates task (where there was no semantic, but phonetic, relationship among the words), are consistent with this conclusion.

## Limitations

Our study has some limitations to consider. The characteristics of the participants selected, healthy university students with high cognitive functioning may limit generalization of the results to other populations. On a technical level, the minimum number of averaged EEG epochs is below the recommended standards. The size of the sample limited a more detailed exploration of the interactions involving sex/gender detected in the P408p component. Loss of participants at follow-up, due to participants declining to participate further or no longer meeting the selection criteria, impeded completion of a robust longitudinal study. Finally, the absence of evaluation of the participants prior to beginning BD prevents any conclusions being reached about causal relationships.

## Conclusion

In summary, this study provides novel information about brain activity during verbal episodic memory in relation to alcohol binge drinking. When retrieving information in a short-term cued-recall paradigm, binge drinkers showed a different pattern of activity in the posterior brain regions. At around 400 ms, the amplitude of the LPC-like component of the ERPs was larger than in controls for new (seen only once) word pairs, which led to an absence of the old/new effect observed in controls. Subsequently, at around 800 ms, the LPN-like component showed a reduced amplitude in response to the same items. No differences associated with alcohol were observed in the late frontal old/new effect. This pattern of brain activity may indicate abnormal functioning of the hippocampal–PPC circuitry responsible for the recognition and recollection of the context of the cue and the generation of the solution to shallowly processed word pairs, to achieve a normal behavioural performance. Although behavioural results were similar for the BDs and the controls in the word pair association task, the BDs showed poor execution of a more demanding test, requiring short-term recall of words cued by semantic information.

## Data Availability

The raw data supporting the conclusion of this article are available at https://osf.io/zftne/.

## References

[B1] Allan K. Robb W. G. K. Rugg M. D. (2000). The effect of encoding manipulations on neural correlates of episodic retrieval. Neuropsychologia 38, 1188–1205. 10.1016/S0028-3932(00)00013-0 10838153

[B2] Allan K. Rugg M. D. (1998). Neural correlates of cued recall with and without retrieval of source memory. NeuroReport 9, 3463–3466. 10.1097/00001756-199810260-00023 9855299

[B3] Almeida-Antunes N. Crego A. Carbia C. Sousa S. S. Rodrigues R. Sampaio A. (2021). Electroencephalographic signatures of the binge drinking pattern during adolescence and young adulthood: A PRISMA-driven systematic review. NeuroImage Clin. 29, 102537. 10.1016/j.nicl.2020.102537 33418172 PMC7803655

[B4] Bai C.-H. Bridger E. K. Zimmer H. D. Mecklinger A. (2015). The beneficial effect of testing: An event-related potential study. Front. Behav. Neurosci. 9, 248. 10.3389/fnbeh.2015.00248 26441577 PMC4584999

[B5] Benedet M. J. Alejandre M. A. (2014). Test de aprendizaje verbal España-Complutense. 2nd Ed. Madrid: Tea Ediciones.

[B6] Carbia C. Cadaveira F. Caamaño-Isorna F. Rodríguez Holguín S. Corral M. (2017). Binge drinking during adolescence and young adulthood is associated with deficits in verbal episodic memory. PLOS ONE 12, e0171393. 10.1371/journal.pone.0171393 28152062 PMC5289570

[B7] Carbia C. López-Caneda E. Corral M. Cadaveira F. (2018). A systematic review of neuropsychological studies involving young binge drinkers. Neurosci. Biobehav. Rev. 90, 332–349. 10.1016/j.neubiorev.2018.04.013 29678643

[B8] Cattell R. B. (1966). The scree test for the number of factors. Multivar. Behav. Res. 1, 245–276. 10.1207/s15327906mbr0102_10 26828106

[B61] Chanraud S. Leroy C. Martelli C. Kostogianni N. Delain F. Aubin H.-J. (2009). Episodic memory in detoxified alcoholics: Contribution of grey matter microstructure alteration. PLoS ONE 4, e6786. 10.1371/journal.pone.0006786 19707568 PMC2728538

[B9] Crews F. T. Nixon K. (2009). Mechanisms of neurodegeneration and regeneration in alcoholism. Alcohol Alcohol 44, 115–127. 10.1093/alcalc/agn079 18940959 PMC2948812

[B10] Cruse D. Wilding E. L. (2009). Prefrontal cortex contributions to episodic retrieval monitoring and evaluation. Neuropsychologia 47, 2779–2789. 10.1016/j.neuropsychologia.2009.06.003 19523968

[B11] Cuenca-Royo A. M. Sánchez-Niubó A. Forero C. G. Torrens M. Suelves J. M. Domingo-Salvany A. (2012). Psychometric properties of the CAST and SDS scales in young adult cannabis users. Addict. Behav. 37, 709–715. 10.1016/j.addbeh.2012.02.012 22386300

[B12] Davis C. J. Perea M. (2005). BuscaPalabras: A program for deriving orthographic and phonological neighborhood statistics and other psycholinguistic indices in Spanish. Behav. Res. Methods 37, 665–671. 10.3758/BF03192738 16629300

[B13] Delis D. C. Kramer J. H. Orber B. A. (1987). California verbal learning test: Adult version manual. San Antonia: The Psychological Corporation.

[B14] Derogatis L. R. (1983). SCL-90-R. Administration, scoring and procedures manual II for the revised version of the SCL-90-R. Baltimore: John Hopkins University Press.

[B15] Dien J. (2010a). Evaluating two-step pca of erp data with geomin, infomax, oblimin, promax, and varimax rotations. Psychophysiology 47, 170–183. 10.1111/j.1469-8986.2009.00885.x 19761521

[B16] Dien J. (2010b). The ERP PCA Toolkit: An open source program for advanced statistical analysis of event-related potential data. J. Neurosci. Methods 187, 138–145. 10.1016/j.jneumeth.2009.12.009 20035787

[B17] Fay S. Isingrini M. Ragot R. Pouthas V. (2005). The effect of encoding manipulation on word-stem cued recall: An event-related potential study. Cogn. Brain Res. 24, 615–626. 10.1016/j.cogbrainres.2005.03.014 16099370

[B18] Ferrando L. Bobes J. Gibert J. Soto M. Soto J. (2000). MINI International neuropsychiatric interview. Spanish version 5.0.0, DSM-IV.

[B19] Folgueira-Ares R. Cadaveira F. Rodríguez Holguín S. López-Caneda E. Crego A. Pazo-Álvarez P. (2017). Electrophysiological anomalies in face–name memory encoding in young binge drinkers. Front. Psychiatry 8, 216. 10.3389/fpsyt.2017.00216 29163235 PMC5671969

[B20] Friedman D. Johnson R. (2000). Event-related potential (ERP) studies of memory encoding and retrieval: A selective review. Microsc. Res. Tech. 51, 6–28. 10.1002/1097-0029(20001001)51:1<6::AID-JEMT2>3.0.CO;2-R 11002349

[B21] Gierski F. Stefaniak N. Benzerouk F. Gobin P. Schmid F. Henry A. (2020). Component process analysis of verbal memory in a sample of students with a binge drinking pattern. Addict. Behav. Rep. 12, 100323. 10.1016/j.abrep.2020.100323 33364330 PMC7752726

[B22] Gratton G. Coles M. G. H. Donchin E. (1983). A new method for off-line removal of ocular artifact. Electroencephalogr. Clin. Neurophysiol. 55, 468–484. 10.1016/0013-4694(83)90135-9 6187540

[B23] Hellerstedt R. Johansson M. (2016). Competitive semantic memory retrieval: Temporal dynamics revealed by event-related potentials. PLOS ONE 11, e0150091. 10.1371/journal.pone.0150091 26901865 PMC4762689

[B24] Hingson R. W. Zha W. (2009). Age of drinking onset, alcohol use disorders, frequent heavy drinking, and unintentionally injuring oneself and others after drinking. Pediatrics 123, 1477–1484. 10.1542/peds.2008-2176 19482757

[B25] Horn J. L. (1965). A rationale and test for the number of factors in factor analysis. Psychometrika 30, 179–185. 10.1007/BF02289447 14306381

[B26] Joel D. Fausto-Sterling A. (2016). Beyond sex differences: New approaches for thinking about variation in brain structure and function. Philos. Trans. R. Soc. B Biol. Sci. 371, 20150451. 10.1098/rstb.2015.0451 PMC4785909 26833844

[B27] Johnson R. Kreiter K. Zhu J. Russo B. (1998). A spatio-temporal comparison of semantic and episodic cued recall and recognition using event-related brain potentials. Cogn. Brain Res. 7, 119–136. 10.1016/S0926-6410(98)00017-2 9774715

[B28] Kang J.-G. Kim M.-S. (2022). Neuropsychological profile of college students who engage in binge drinking. Front. Psychol. 13, 873654. 10.3389/fpsyg.2022.873654 35496236 PMC9051325

[B29] Kelly A. T. Mozayani A. (2012). An overview of alcohol testing and interpretation in the 21st century. J. Pharm. Pract. 25, 30–36. 10.1177/0897190011431149 22215644

[B30] Keselman H. J. Wilcox R. R. Lix L. M. (2003). A generally robust approach to hypothesis testing in independent and correlated groups designs. Psychophysiology 40, 586–596. 10.1111/1469-8986.00060 14570166

[B31] Kim H. (2019). Neural correlates of explicit and implicit memory at encoding and retrieval: A unified framework and meta-analysis of functional neuroimaging studies. Biol. Psychol. 145, 96–111. 10.1016/j.biopsycho.2019.04.006 31034858

[B32] Lannoy S. Billieux J. Dormal V. Maurage P. (2019). Behavioral and cerebral impairments associated with binge drinking in youth: A critical review. Psychol. Belg. 59, 116–155. 10.5334/pb.476 31328014 PMC6625552

[B33] Lees B. Mewton L. Stapinski L. A. Squeglia L. M. Rae C. D. Teesson M. (2019). Neurobiological and cognitive profile of young binge drinkers: A systematic review and meta-analysis. Neuropsychol. Rev. 29, 357–385. 10.1007/s11065-019-09411-w 31512192 PMC7231524

[B34] Maurage P. Lannoy S. Mange J. Grynberg D. Beaunieux H. Banovic I. (2020). What we talk about when we talk about binge drinking: Towards an integrated conceptualization and evaluation. Alcohol Alcohol 55, 468–479. 10.1093/alcalc/agaa041 32556202

[B35] Mecklinger A. Rosburg T. Johansson M. (2016). Reconstructing the past: The late posterior negativity (LPN) in episodic memory studies. Neurosci. Biobehav. Rev. 68, 621–638. 10.1016/j.neubiorev.2016.06.024 27365154

[B36] Meda S. A. Hawkins K. A. Dager A. D. Tennen H. Khadka S. Austad C. S. (2018). Longitudinal effects of alcohol consumption on the hippocampus and parahippocampus in college students. Biol. Psychiatry Cogn. Neurosci. Neuroimaging 3, 610–617. 10.1016/j.bpsc.2018.02.006 29680476 PMC6062479

[B37] National Institute on Alcohol Abuse and Alcoholism (2016). Drinking levels defined. Available at: https://www.niaaa.nih.gov/alcohol-health/overview-alcohol-consumption/moderate-binge-drinking.

[B38] Nuwer M. R. Comi G. Emerson R. Fuglsang-Frederiksen A. Guérit J.-M. Hinrichs H. (1998). IFCN standards for digital recording of clinical EEG. International Federation of Clinical Neurophysiology. Electroencephalogr. Clin. Neurophysiol. 106, 259–261. 10.1016/S0013-4694(97)00106-5 9743285

[B39] Palacio N. Cardenas F. (2019). A systematic review of brain functional connectivity patterns involved in episodic and semantic memory. Rev. Neurosci. 30, 889–902. 10.1515/revneuro-2018-0117 31323012

[B40] Persson J. Nyberg L. (2000).Conjunction analysis of cortical activations common to encoding and retrieval. Microsc. Res. Tech. 51, 39–44. 10.1002/1097-0029(20001001)51:1<39::AID-JEMT4>3.0 11002351

[B41] Pfefferbaum A. Kwon D. Brumback T. Thompson W. K. Cummins K. Tapert S. F. (2018). Altered brain developmental trajectories in adolescents after initiating drinking. Am. J. Psychiatry 175, 370–380. 10.1176/appi.ajp.2017.17040469 29084454 PMC6504929

[B60] Poznyak V. Rekve D. (2018). Global status report on alcohol and health 2018. World Health Organization. Available at: https://www.who.int/publications/i/item/9789241565639 (Accessed March 27, 2022).

[B42] Ranganath C. Ritchey M. (2012). Two cortical systems for memory-guided behaviour. Nat. Rev. Neurosci. 13, 713–726. 10.1038/nrn3338 22992647

[B43] Rivas-Fernández M. Á. Galdo-Álvarez S. Zurrón M. Díaz F. Lindín M. (2020). Spatiotemporal pattern of brain electrical activity related to immediate and delayed episodic memory retrieval. Neurobiol. Learn. Mem. 175, 107309. 10.1016/j.nlm.2020.107309 32890759

[B44] Rugg M. D. Curran T. (2007). Event-related potentials and recognition memory. Trends Cogn. Sci. 11, 251–257. 10.1016/j.tics.2007.04.004 17481940

[B45] Saunders J. B. Aasland O. G. Babor T. F. De La Fuente J. R. Grant M. (1993). Development of the alcohol use disorders identification test (AUDIT): WHO collaborative project on early detection of persons with harmful alcohol consumption-II. Addiction 88, 791–804. 10.1111/j.1360-0443.1993.tb02093.x 8329970

[B46] Schweinsburg A. D. McQueeny T. Nagel B. J. Eyler L. T. Tapert S. F. (2010). A preliminary study of functional magnetic resonance imaging response during verbal encoding among adolescent binge drinkers. Alcohol 44, 111–117. 10.1016/j.alcohol.2009.09.032 20113879 PMC2845466

[B47] Schweinsburg A. D. Schweinsburg B. C. Nagel B. J. Eyler L. T. Tapert S. F. (2011). Neural correlates of verbal learning in adolescent alcohol and marijuana users. Addiction 106, 564–573. 10.1111/j.1360-0443.2010.03197.x 21134014 PMC3423457

[B48] Shiffman S. Waters A. Hickcox M. (2004). The nicotine dependence Syndrome Scale: A multidimensional measure of nicotine dependence. Nicotine Tob. Res. 6, 327–348. 10.1080/1462220042000202481 15203807

[B49] Silins E. Horwood L. J. Najman J. M. Patton G. C. Toumbourou J. W. Olsson C. A. (2018). Adverse adult consequences of different alcohol use patterns in adolescence: An integrative analysis of data to age 30 years from four australasian cohorts. Addiction 113, 1811–1825. 10.1111/add.14263 29749666

[B50] Smith J. L. De Blasio F. M. Iredale J. M. Matthews A. J. Bruno R. Dwyer M. (2017). Verbal learning and memory in cannabis and alcohol users: An event-related potential investigation. Front. Psychol. 8, 2129. 10.3389/fpsyg.2017.02129 29276495 PMC5727079

[B51] Smith J. L. Iredale J. M. Mattick R. P. (2016). Sex differences in the relationship between heavy alcohol use, inhibition and performance monitoring: Disconnect between behavioural and brain functional measures. Psychiatry Res. Neuroimaging 254, 103–111. 10.1016/j.pscychresns.2016.06.012 27399307

[B52] Spear L. P. (2018). Effects of adolescent alcohol consumption on the brain and behaviour. Nat. Rev. Neurosci. 19, 197–214. 10.1038/nrn.2018.10 29467469

[B53] Squire L. R. Genzel L. Wixted J. T. Morris R. G. (2015). Memory consolidation. Cold Spring Harb. Perspect. Biol. 7, a021766. 10.1101/cshperspect.a021766 26238360 PMC4526749

[B54] Staresina B. P. Wimber M. (2019). A neural chronometry of memory recall. Trends Cogn. Sci. 23, 1071–1085. 10.1016/j.tics.2019.09.011 31672429

[B55] Sullivan E. V. (2017). Contributions to understanding the neuropsychology of alcoholism: An INS legacy. J. Int. Neuropsychol. Soc. 23, 843–859. 10.1017/S1355617717000674 29198270 PMC8356744

[B56] Townshend J. M. Duka T. (2002). Patterns of alcohol drinking in a population of young social drinkers: A comparison of questionnaire and diary measures. Alcohol Alcohol 37, 187–192. 10.1093/alcalc/37.2.187 11912076

[B57] Walker C. D. Kuhn C. M. Risher M.-L. (2021). The effects of peri-adolescent alcohol use on the developing hippocampus. Int. Rev. Neurobiol. 160, 251–280. 10.1016/bs.irn.2021.08.003 34696875

[B62] White A. M. Matthews D. B. Best P. J. (2000). Ethanol, memory, and hippocampal function: A review of recent findings. Hippocampus 10, 88–93. 10.1002/(SICI)1098-1063(2000)10:1<88::AID-HIPO10>3.0.CO;2-L 10706220

[B58] Wilding E. L. Ranganath C. (2012). “Electrophysiological correlates of episodic memory processes,” in The Oxford handbook of event-related potential components. Editors Luck J. Kappenman E. S. (Oxford University Press), 373–395.

[B59] Wilding E. L. Rugg M. D. (1996). An event-related potential study of recognition memory with and without retrieval of source. Brain 119, 889–905. 10.1093/brain/119.3.889 8673500

