# Corrigendum: Neurocognitive effects of binge drinking on verbal episodic memory. An ERP study in university students

**DOI:** 10.3389/fphar.2023.1362687

**Published:** 2024-01-09

**Authors:** Socorro Rodríguez Holguín, Rocío Folgueira-Ares, Alberto Crego, Eduardo López-Caneda, Montserrat Corral, Fernando Cadaveira, Sonia Doallo

**Affiliations:** ^1^ Department of Clinical Psychology and Psychobiology, Universidade de Santiago de Compostela (USC), Santiago de Compostela, Spain; ^2^ Psychological Neuroscience Laboratory (PNL), Research Center in Psychology (CIPsi), School of Psychology, University of Minho, Campus de Gualtar, Gualtar, Portugal

**Keywords:** alcohol, binge drinking, university students, verbal episodic memory, event-related brain potentials (ERP)

In the published article, there was an error in the **Data availability statement**. It was stated that “The raw data supporting the conclusions of this article will be made available by the authors, without undue reservation.” The correct **Data availability statement** appears below.

## Data availability statement

The raw data supporting the conclusion of this article are available at https://osf.io/zftne/.

The authors apologize for this error and state that this does not change the scientific conclusions of the article in any way. The original article has been updated.

