# Supplementary material for: Neurocognitive effects of binge drinking on verbal episodic memory. An ERP study in university students
Source: Front Pharmacol. 2023 Feb 7;14:1034248. doi: 10.3389/fphar.2023.1034248 (PMC9941344; doi:10.3389/fphar.2023.1034248)
Supplement: Supplementary file 1 [file DataSheet1.PDF]

Supplementary material

Neurocognitive effects of binge drinking on verbal episodic memory. An ERP study in university students.

Socorro Rodríguez Holguín, Rocío Folgueira-Ares, Alberto Crego, Eduardo López-Caneda, Montserrat Corral, Fernando Cadaveira, Sonia Doallo.

1. Sex-disaggregated data

Sex was considered in a preliminary analysis to decide its inclusion in the statistical design. This factor showed interaction with Group or Stimulus type only on one variable, the ERP component TF03SF1 (P408p), so it was included in the statistical design only to address this dependent variable.

However, since sex-disaggregated data could be of interest for meta-analytic reviews of the research, they are included here for the dependent variables included in the study (demographics, alcohol consumption, ERP components and behavioural performance).

a) Demographic and consumption characteristics

Table S.1. Demographic and substance use characteristics of the sample (mean ± standard deviation) for each group (controls and binge drinkers) and sex (females and males).

	**Controls**		**Binge drinkers**	
	**Females**	**Males**	**Females**	**Males**
N	18	20	17	15
Age [range 18-19]	18.5 ± 0.3	18.4 ± 0.3	18.5 ± 0.3	18.3 ± 0.3
Age of drinking onset	17.25 ± 1.76	16.42 ± 1.00	14.82 ± 1.19	15.67 ± 1.11
Total grams of alcohol in a standard drinking episode	8.75 ± 11.70	12.25 ± 14.94	105.88 ± 23.85	113.50 ± 36.45
Speed of consumption: drinks/hour	0.24 ± 0.34	0.26 ± 0.35	3.18 ± 0.39	3.60 ± 0.99
Estimated BAC in a standard drink episode[a,]	0.005 ± 0.01	0.007 ± 0.01	0.26 ± 0.08	0.19 ± 0.07
Percentage of times became drunk when drinking	0.00 ± 0.00	0.50 ± 2.24	40.00 ± 21.14	39.33 ± 29.81
Total AUDIT score [range]	0.47 ± 0.80 [0-3]	1.35 ± 1.78 [0-6]	9.47 ± 2.85 [4-14]	8.93 ± 2.12 [5-13]
N regular tobacco smokers[b]	0	0	1	3
SCL-90-R - GSI (percentile scores)	21.39 ± 20.42	23.33 ± 23.58	36.25 ± 33.29	32.00 ± 26.98

[a]gr/dL; [b]Daily (max. 5 cigarettes/day); SCL-90-R - GSI: Symptom checklist-90-revised - global severity index; AUDIT: Alcohol use disorders identification test.

b) ERP components amplitude

Table S.2. Mean (standard deviation) amplitude (μV) of the ERP components for each group (controls and binge drinkers), sex (females and males) and type of stimulus (old and new word pairs, OP/NP).

Factor / ERP component	Controls				Binge drinkers			
	Females		Males		Females		Males	
	OP	NP	OP	NP	OP	NP	OP	NP
TF03SF1 / P408p	0.76 (6.53)	-1.86 (4.96)	2.69 (5.42)	1.81 (5.51)	4.77 (5.64)	3.70 (6.11)	1.16 (6.62)	2.07 (6.39)
TF02SF1 / N812p	-4.89 (8.26)	-8.67 (7.42)	-2.32 (5.87)	-5.58 (6.96)	-4.68 (6.82)	-6.51 (5.44)	-3.33 (7.57)	-3.09 (6.91)
TF01SF2 / P1656a	1.31 (1.91)	2.60 (2.05)	1.45 (2.16)	2.06 (1.96)	1.54 (2.89)	2.73 (2.27)	1.66 (1.60)	2.39 (2.06)

c) Behavioural performance

Table S.3. Descriptive data on the behavioural measures. Percentage of hits in the verbal memory task (old and new word pairs, OP/NP) and scores in the Short-term cued-recall measure of the TAVEC. Mean (standard deviation) for each group and sex.

	Controls				Binge drinkers			
	Females		Males		Females		Males	
	OP	NP	OP	NP	OP	NP		
Verbal memory task. % hits	93.52 (6.54)	67.36 (16.39)	90.00 (9.57)	66.15 (17.57)	92.77 (6.55)	66.42 (15.24)	91.53 (6.37)	69.03 (13.22)
TAVEC Short-term cued-recall score [range 0-16]	14.33 (1.75) [10-16]		13.55 (1.76) [10-16]		13.00 (1.77) [11-16]		12.80 (1.97) [10-16]	

2. Follow-up study

A subsample of the subjects could de followed up two years later. It was too small to achieve enough statistical power, but the data were analysed for exploratory purposes and are presented here.

Participants

The subjects included in the study were contacted after approximately two years (22.5 ± 1.2 months) and asked if they would participate in a follow-up study. Thirty-two of the 70 subjects agreed to participate and met the same inclusion and exclusion criteria: 11 binge drinkers (6 females) and 21 controls (10 females). The demographics and substance use characteristics of this subset, at the time of both evaluations are included in Table S.4.

Table S.4. Demographics and substance use characteristics of the subjects participating in the 2nd evaluation (mean ± standard deviation)

	Controls		Binge drinkers	
	**1st evaluation**	**2nd evaluation**	**1st evaluation**	**2nd evaluation**
N (females)	21 (10)		11 (6)	
Age [range]	18.5 ± 0.3 [18-19]	20.4 ± 0.3 [20-21]	18.3 ± 0.3 [18-19]	20.3 ± 0.3 [20-21]
Age of drinking onset*	17.00 ± 1.71		15.27 ± 0.90	
Total grams of alcohol in a standard drinking episode*	8.57 ± 9.76	24.64 ± 23.46	125.68 ± 25.79	107.68 ± 47.65
Speed of consumption: drinks/hour*	0.25 ± 0.32	0.43 ± 0.48	3.73 ± 0.79	2.14 ± 1.14
Estimated BAC in a standard drink episode[a]*	0.004 ± 0.001	0.02 ± 0.03	0.28 ± 0.07	0.22 ± 0.19
Percentage of times became drunk when drinking*	0.00 ± 0.00	16.43 ± 34.61	47.73 ± 25.04	82.73 ± 25.23
Total AUDIT score* [range]	0.81 ± 1.17 [0.4]	1.24 ± 1.18 [0-3]	10.00 ± 2.72 [5-14]	8.09 ± 2.12 [5-12]
N regular tobacco smokers[b]	0	0	0	1
SCL-90-R - GSI percentile scores	27.11 ± 26.05	26.90 ± 25.37	35.91 ± 30.81	37.27 ± 28.23

[a]gr/dL (calculated using the classic Widmark formula (Widmark, 1932, see in Kelly and Mozayani, 2012); [b]Daily; SCL-90-R - GSI: Symptom checklist-90-revised - global severity index; AUDIT: Alcohol use disorders identification test
* Student t-test (CN vs. BD), $p < 0.05$ (both at the 1st and the 2nd assessment)

Alcohol consumption variables, at the time of the first evaluation were compared between those subjects assessed at follow-up and those who abandoned the study; the results are shown in Table S.5.

Table S.5. Comparsion of the alcohol consumption characteristics (at the 1st evaluation) between those subjects assessed at follow-up and those who abandoned the study (p-values from the Student t Test).

	Controls	Binge drinkers
Age of drinking onset	n.s.	n.s.
Total grams of alcohol in a standard drinking episode	n.s.	.025*
Speed of consumption: drinks/hour	n.s.	n.s.
Estimated BAC in a standard drink episode[a]	n.s.	.016**
Percentage of times became drunk when drinking	n.s.	n.s.
Total AUDIT score	n.s.	n.s.
SCL-90-R: GSI percentile scores	n.s.	n.s.

*Assessed (125.68 gr) > abandoned (100.95 gr);**assessed (0.028 gr/dL) > abandoned (0.020 gr/dL)

Procedure and EEG data acquisition and processing

The procedure (tasks, EEG recording and processing) was the same as in the first evaluation.

Statistical analysis

Data from the 2nd evaluation were examined by means of an Evaluation x Group x Type of stimulus design, applied to the ERP and behavioural dependent variables.

Results

ERP components

Analysis including the 2nd evaluation (Evaluation x Type of stimulus x Group) yielded the following results:

Evaluation was significant only for N812p, (TWJt/c(1.0,20.2) = 6.66, p = 0.0149, MSe = 29.45), with smaller amplitude in the 2nd (-2.12 µV) than the 1st (-4.78 µV) evaluation. This factor did not interact with Type of Stimulus or Group for any of the four ERP components.

Type of stimulus (old/new effect) was significant for the three ERP components: P408p (TWJt/c(1.0,18.9) = 6.38, p = 0.0213, MSe = 6.97; OP > NP); N812p (TWJt/c(1.0,26.3) = 9.31, p = 0.0049, MSe = 10.72; OP < NP), and P1656a (TWJt/c(1.0,19.2) = 29.78, p = 0.000052, MSe = 1.66; OP < NP).

Group did not have a main effect on any of the ERP components, but the Type of stimulus x Group interaction remained significant for N812p (TWJt/c(1.0,26.3) = 8.04, p = 0.0087, MSe = 10.72) and emerged for P1656a (TWJt/c(1.0,19.2) = 5.78, p = 0.027, MSe = 1.66).

Behavioural performance

In the 2nd evaluation, results evidenced better performance for OP (94.79 %) than NP (73.83 %) (F(1,30) = 80.403, p < .0001, η2p = .728), and no Group effects or interactions. As for the analysis including both the evaluations, besides the main effect of Type of stimulus (F(1,30) = 117.780, p < .0001, η2p = .797), Evaluation also had an effect (F(1,30) = 23.792, p < .0001, η2p = .442), with better performance in the 2nd evaluation, and no effects including the Group factor.

Regarding the TAVEC measure (short-term cued-recall score) at the 2nd evaluation, there were no differences in this variable between the groups. Analysis including both evaluations revealed a significant improvement in performance (F(1,30) = 11.796, p = .002, η2p = .282) and also an Evaluation x Group interaction (F(1,30) = 4.281, p = .047, η2p = .125), showing that differences between groups in the 1st evaluation disappeared in the 2nd evaluation, and also that the improvement between evaluations only occurred in the BD group, whereas the performance of the CN group was similar in the 1st and 2nd evaluations.

Descriptive statistics of behavioural performance are summarized in Table S.6.

Table S.6. Descriptive data on the behavioural measures of subjects who undertook the two evaluations. Percentage of hits in the verbal memory task (old and new word pairs, OP/NP) and scores in the Short-term cued-recall measure of the TAVEC. Mean (standard deviation) for each group and evaluation.

	**Evaluation**	**Controls**		**Binge drinkers**	
		**OP**	**NP**	**OP**	**NP**
Verbal memory task % hits	1st evaluation	91.96 (7.83)	66.37 (16.68)	91.29 (5.95)	66.67 (12.04)
	2nd evaluation	94.74 (7.26)	74.31 (16.54)	94.89 (4.69)	72.92 (15.34)
TAVEC Short-term cued-recall score [range 0-16]	1st evaluation	14.10 (1.81) [10-16]		12.64 (1.63) [10-15]	
	2nd evaluation	14.52 (1.78) [10-16]		14.36 (1.86) [11-16]	

Discussion

Regarding the results of the follow-up, as stated above, the follow-up sample was too small to enable these results to be considered as anything more than exploratory. The two-year interval between evaluations only had a significant effect on N812p, in which the observed reduction in amplitude was similar for both types of stimuli and both groups. The old/new effects persisted, but interactions with group lost significance for P408p and emerged for P1656a.

The small number of subjects precludes drawing firm conclusions, but difference in results from the first assessment (disappearance of the group effect in one ERP component but appearance in another) would be consistent with the expected potential change in the effects of binge drinking on verbal episodic memory over time, and highlight the importance of follow-up studies. It will be of interest to confirm whether anomalies in the late frontal component, absent in the first evaluation, actually emerge after two years of maintenance of the BD pattern of alcohol consumption.

Conclusions

Exploratory follow-up of a small subsample indicated that persistence of BD consumption could lead to the emergence of anomalies in frontal activity associated with postretrieval monitoring processes. This result underlines the need for follow-up studies to further investigate this evolution.
